# Perceived Discrimination Is an Independent Risk Factor for Suicidal Ideation among Sexual and Gender Minorities in Nepal

**DOI:** 10.1371/journal.pone.0159359

**Published:** 2016-07-20

**Authors:** Verena Kohlbrenner, Keshab Deuba, Deepak Kumar Karki, Gaetano Marrone

**Affiliations:** 1 Karolinska Institutet, Stockholm, Sweden; 2 Public Health and Environment Research Center, Kathmandu, Nepal; 3 Department of Public Health Sciences, Karolinska Institutet, Stockholm, Sweden; 4 Department of Health Science, Nobel College, Pokhara University, Kathmandu, Nepal; 5 Department of Infectious Disease, Karolinska Hospital, Stockholm, Sweden; Medical University of Vienna, AUSTRIA

## Abstract

Sexual and gender minorities experience an elevated burden of suicidality compared with the general population. Still, little is known about that burden and the factors generating it in the context of low- and middle-income countries. The present study assessed the prevalence of suicidal ideation, planned suicide, and attempted suicide among men who have sex with men (MSM) and transgender people (TG) in Nepal, and examined the association of perceived discrimination on the basis of sexual orientation with suicidal ideation and with attempted suicide. Data were obtained from a surveillance survey among MSM and TG in Nepal in 2012. A sample of 400 MSM and TG, recruited using respondent-driven sampling, completed a structured face-to-face interview. Throughout their lifetime, 26.8% of the participants had experienced suicidal ideation, 12.0% had made a suicide plan, and 9.0% had attempted suicide. In particular, more TG than MSM had experienced suicidal ideation (39.8% vs. 21.3%), had made a suicide plan (19.5% vs. 8.9%), and had attempted suicide (15.3% vs. 6.4%). Overall, the odds of having experienced suicidal ideation was significantly higher among the 38.3% of participants who had perceived discrimination based on their sexual orientation (AOR: 3.17; 95% CI: 1.83–5.48). Moreover, the odds of suicidal ideation was significantly higher as the extent of perceived discrimination increased (AOR: 1.35; 95% CI: 1.15–1.60). However, the odds of attempted suicide was not significantly associated with perceived discrimination (AOR: 1.40; 95% CI: 0.62–3.15). The findings highlight perceived discrimination as an independent risk factor for suicidal ideation. Future suicide prevention programs should target sexual and gender minorities and include elements focusing on discrimination.

## Introduction

Sexual and gender minorities experience numerous health disparities compared with the general population. The latest Institute of Medicine (IOM) report points to health disparities regarding HIV, sexually transmitted infections, and certain forms of cancer; health disparities regarding depression, anxiety disorders, and suicidality; and health disparities regarding stigma, violence, and discrimination [1]. Although the wide range of health disparities is well documented, large gaps still exist in our understanding of their formation, and of the contextual factors that influence sexual and gender minorities over their life course [1]. According to the National Institute of Health (NIH) of the United States, much of the current research on sexual and gender minorities focuses on behavioral and social science, HIV, mental health, and substance abuse and little attention is directed towards several crucial health areas, including suicidality [2]. Notably, in the past decade there were at least ten times fewer suicide and suicidality related citations compared with HIV related citations in the scientific literature on health among sexual and gender minorities [3]. This indicates the substantial need to scale up research on suicidality among this particular population.

The lifetime prevalence of attempted suicide is around four times higher among gay and bisexual men compared with heterosexual men, according to a meta-analytic review [4]. In general, there is a wide spectrum of established risk factors for suicide, including social disadvantages, alcohol and substance use disorders, mental disorders, lack of social support, and stressful life events [5]. One possible explanation for the elevated prevalence of attempted suicide among sexual and gender minorities is the fact that within this spectrum, certain risk factors are unique to sexual and gender minorities, such as stressful life events resulting from discrimination based on sexual orientation [6,7]. It is anticipated that these risk factors are additive to the risk factors that accrue in the general population, and therefore lead to a higher risk of suicide among sexual and gender minorities [8].

Despite the diversity of socio-demographic and psychosocial characteristics within sexual and gender minorities, their members often share similar experiences related to discrimination based on sexual orientation [9,10]. The experience of discrimination is considered a stressor with a broad potential impact on health. Discrimination creates a stressful social environment and a stressful social environment can lead to negative health outcomes [8]. Perceived discrimination on the basis of sexual orientation, in particular, was found to be a risk factor for suicidality among sexual and gender minorities in high-income countries [11,12,13]. A school-based survey from the United States, for instance, revealed that perceived discrimination accounted for an increased risk of suicidal ideation among gay and bisexual male youth [13]. The extent to which these findings can be transferred to sexual and gender minorities in low- and middle-income countries remains unknown: while 75% of all global suicides occur in low-and middle-income countries [14], almost all related research has been conducted in high-income countries [15].

To address these gaps in our knowledge, in the present study we examined the lifetime prevalence of suicidal ideation, planned suicide and attempted suicide among men who have sex with men (MSM) and male to female transgender people (TG) in Nepal, a low-income country located in the WHO region of South-East Asia. Notably, almost 40% of all global suicides occur in this region [14]. Furthermore, we explored the association between perceived discrimination on the basis of sexual orientation and suicidality (suicidal ideation and attempted suicide). We assumed that perceived discrimination, and its extent, would be associated with a higher prevalence of suicidality, independent of potential confounders and covariates. Nepal is one of the most progressive countries within South-East Asia regarding rights of sexual and gender minorities. After the end of a decade long armed conflict, a new constitution has been formulated and recently ratified, and this includes explicit protections for sexual and gender minorities from discrimination, violence, and abuse. Nevertheless, human rights violations and discrimination against sexual and gender minorities are still common; this is in part because Nepal’s society is structured through strong religious beliefs and conservative values that condemn sexual orientations other than heterosexual [16]. Using snowball sampling, recent research among gay and bisexual men in Nepal found a prevalence of 46.9% for suicidal ideation [17]. In the same study, 69.3% of the participants had experienced verbal, physical or sexual abuse, leading to the hypothesis that a high burden of perceived discrimination based on sexual orientation might be present. In the present study, we explored the potential association between these findings.

## Methods

We obtained cross-sectional data from the fourth round of the Integrated Biological and Behavioral Surveillance surveys among MSM and TG in Nepal [18]. The surveillance surveys were established in Nepal in 1990, primarily to monitor the HIV epidemic among populations at high risk; they were conducted every two to three years thereafter. From September 10th to October 16th 2012, 400 MSM and TG residing in Kathmandu Valley were recruited using respondent-driven sampling (RDS). The sampling process was launched by recruiting eight initial seeds, differing in age, ethnicity, geographical distribution, and involvement in transactional sex. To be eligible for the survey, participants had to be 16 years of age or above, self-identified as either MSM or male to female TG (“How do you identify yourself on the basis of your sexual orientation or behavior? How do you identify yourself on the basis of gender?”), and reported to have had sexual relations with another biological male in the past year (“Have you had anal or oral sex with a male or male to female transgender person in the last 12 months?”).

Data were collected through face-to-face interviews after briefing, checking eligibility and obtaining verbal informed and witnessed consent. Interviewers used a structured interview schedule and had completed an intensive training beforehand, covering objectives and content of the survey, characteristics of the MSM and TG populations and rapport-building techniques. The interviews were followed by a clinical examination. After participation, participants were encouraged to recruit up to three other MSM or TG from their social networks and were given three recruitment coupons each. The coupons were uniquely coded to monitor the recruitment process and referred participants had to present their coupon at arrival. Small financial incentives were given for participation and for each additional participant recruited. The target sample size of 400 MSM and TG was calculated based on the guidelines for repeated behavioral surveys in populations at risk of HIV [19], and recruitment through RDS continued until this total was reached.

### Measures

Information on suicidality, perceived discrimination and most potential confounders and covariates was obtained via the interview. HIV-infection status was obtained via lab testing of the blood sample drawn during the clinical examination.

#### Suicidality

The assessment of suicidality was based on three dichotomous items asking for suicidal ideation (“Have you ever felt so low you thought a lot about committing suicide?”), planned suicide (“Have you ever made a plan to commit suicide?”), and attempted suicide (“Have you ever attempted suicide?”). Only participants who had reported suicidal ideation were further asked about planned and attempted suicide, since suicidality can be conceptualized as a continuum starting with suicidal ideation, extending to planned suicide, and ending with attempted suicide [20]. In line with this, suicidal ideation is considered the main outcome within the present study. Although suicidal ideation might not appear to be a stable predictor for suicide [21], it is a direct precursor and the average time period between the onset of suicidal ideation and attempting suicide is known to be short [22,23]. Therefore, focusing on suicidal ideation allows designing prevention strategies that engage at the earliest stage of suicidality. Nonetheless, a prior suicide attempt constitutes the single most important risk factor for committed suicide [15]. Therefore, attempted suicide was also examined in detail.

#### Perceived discrimination

A modified version of the Experience of Discrimination tool (EOD) was implemented [24,25,26]. Participants were asked about discrimination in relation to their sexual orientation regarding eight different situations (“Have you ever experienced discrimination, been prevented from doing something, or been hassled or made to feel inferior in any of the following situations because of your sexual orientation?”). The eight situations referred to school, getting hired, work, getting housing, getting medical care, getting service in a store or restaurant, public settings, and to interactions with the police or other security personnel. In addition to a dichotomous variable indicating perceived discrimination in any of the eight situations, a discrete numerical variable was calculated based on the number of situations in which discrimination was perceived, indicating the extent of perceived discrimination. Reliability and validity indices for the original EOD version comprise high scale reliability (Cronbach’s alpha = .74) and the highest correlation (r = .79) with an underlying discrimination construct compared with other self-report discrimination measures [26].

#### Potential confounders and covariates

Factors having an established or theoretical association with suicidality were taken into consideration. Self-reported gender identity, age, religion, marital status, living situation, education, occupation, income, and sexual experience were recorded. Measures on sexual experience included age at first sex, whether it was consensual, gender of the first sexual partner, and engagement in transactional sex. Further, information on alcohol use in the past month, illicit drug use in the past year, and HIV-infection status was acquired. The recording of psychosocial characteristics included measures on depression, social support, and familial factors. The Center for Epidemiological Studies Depression scale (CES-D) was used to assess depression [27]. It consists of 20 items asking for the presence of depressive symptoms in the past week, rated on a scale ranging from 0 (“rarely or none of the time”) to 3 (“most or all of the time”). A validated cut-off score of 16 or higher was applied, suggesting significant depressive symptoms [28]. Social support was measured through the Social Support Questionnaire (SSQ) short form [29]. It includes six items assessing the number of supportive persons who can be counted on for different situations, ranging from 0 to 9, and the satisfaction associated with the available support, ranging from 1 (“very dissatisfied”) to 6 (“very satisfied”). Two scores were calculated, the SSQ number score (SSQN) and the SSQ satisfaction score (SSQS). The assessment of familiar factors included three dichotomous items asking for family openness (“Is there at least someone in your immediate family that you can talk openly with about your homosexual or bisexual behavior?”), forced marriage (“Does your family force you into marriage with female?”), and family rejection (“Does your family force you to live outside of home because of your sexual orientation or behavior?”).

### Statistical analysis

Descriptive analyses included frequencies for categorical variables and mean, standard deviation, and range for continuous variables. Based on the network characteristics of each participant, RDS-II estimates were used for the calculation of probability weights and RDS-adjusted prevalence [30]. Further, homophily estimates were calculated, measuring the likelihood of participants recruiting other participants with the same characteristic. A value of 1 represents random recruitment. Values below 1 represent a lower likelihood to recruit someone with the same characteristic and values above 1 represent a higher likelihood to recruit someone with the same characteristic. Since performing regression analysis on RDS data is not yet sufficiently developed [31], only descriptive statistics were conducted with RDS-adjustment.

Multiple logistic regression was used to examine the association between the outcome variables (suicidal ideation and attempted suicide) and perceived discrimination under consideration of potential confounders and covariates. The prevalence of the outcome was compared between the categories of each variable using Chi-square tests for categorical and t-tests for continuous variables. Variables presenting a p-value ≤ .20 in the bivariate analysis were included in the multiple logistic regressions. The final models were compiled using backward stepwise elimination with removing variables presenting a p-value > .10 from the saturated model. Following this approach, three multiple logistic regression models were computed in primary analysis. The first model explored the association between suicidal ideation and perceived discrimination among all participants. The second model explored the association between suicidal ideation and the extent of perceived discrimination among the participants who had perceived discrimination on the basis of sexual orientation. The third model explored the association between attempted suicide and perceived discrimination among all participants. In secondary analysis, the same multiple logistic regression models were computed separately for MSM and TG, resulting in six additional models. All final models contained no variables with missing data and the variance inflation factors (VIF) indicated no problematic multicollinearity (all VIF-values ≤ 1.29). The final models were further tested for interactions. None of the interaction terms were found to be significant, thus they were excluded from the final models. All statistical tests were based on a significance level of a p-value ≤ .05 and confidence intervals (CI) were set at 95% confidence level.

### Ethical statement

Ethical approval was obtained prior to the fieldwork from the national ethics committee, the Nepal Health Research Council, and the survey was conducted in accordance with ethical and human rights standards. Verbal informed and witnessed consent was obtained from all participants following a detailed description of the research and objectives, possible risks and benefits, their rights as participants and the confidentiality policy. Verbal informed and witnessed consent was chosen over written informed consent to guarantee that all participants received the full information and illiterate participants were not at a disadvantage. In order to ensure confidentiality, all participants were provided a unique identification number and no personal identifiers were collected.

The Nepal Health Research Council was informed and approved that minors would be included in the survey and would be asked for verbal informed and witnessed consent. Obtaining informed consent on behalf of minors from the next of kin, caretakers, or guardians was precluded due to the prevalent condemnation and stigma regarding sexual orientations other than heterosexual in Nepal [16].

## Results

In total, 400 participants enrolled, of which 70.5% (RDS-adjusted estimate: 83.5%; 95% CI: 75.2–89.5) identified themselves as MSM and 29.5% (RDS-adjusted estimate: 16.5%; 95% CI: 10.5–24.8) as TG. Frequencies for suicidality are displayed in Table 1. Overall, 26.8% (RDS-adjusted estimate: 14.2%; 95% CI: 9.8–20.1) of the participants had experienced suicidal ideation, 12.0% (RDS-adjusted estimate: 6.0%; 95% CI: 3.4–10.3) had planned to commit suicide and 9.0% (RDS-adjusted estimate: 5.0%; 95% CI: 2.6–9.3) had attempted suicide. Suicidality was higher among TG than MSM in all three measures (all p-values ≤ .005). Frequencies for perceived discrimination on the basis of sexual orientation regarding the different situations are displayed in Table 2. Discrimination on the basis of sexual orientation in at least one situation was reported by 38.3% of all participants (RDS-adjusted estimate: 22.4%; 95% CI: 15.4%-31.5%) and higher among TG than MSM (75.4% vs. 22.7%, p < .001).

**Table 1:** Suicidality among MSM and TG in Nepal in 2012.

Characteristic	MSM (n = 282)	TG (n = 118)	Overall (n = 400)		
	Frequency (%)	Frequency (%)	Frequency (%)	RDS-adjusted % (95% CI)	Homophily
Suicidal ideation***					
No	222 (78.7)	71 (60.2)	293 (73.3)	85.8 (79.9–90.2)	1.02
Yes	60 (21.3)	47 (39.8)	107 (26.8)	14.2 (9.8–20.1)	
Planned suicide**					
No	257 (91.1)	95 (80.5)	352 (88.0)	94.0 (89.7–96.6)	1.15
Yes	25 (8.9)	23 (19.5)	48 (12.0)	6.0 (3.4–10.3)	
Attempted suicide**					
No	264 (93.6)	100 (84.7)	364 (91.0)	95.0 (90.7–97.4)	1.06
Yes	18 (6.4)	18(15.3)	36 (9.0)	5.0 (2.6–9.3)	

*Note*. MSM = men who have sex with men; TG = male to female transgender people; RDS = respondent-driven sampling; CI = confidence interval

** p < .01;

*** p < .001

**Table 2:** Association between perceived discrimination on the basis of sexual orientation and suicidal ideation.

Perceived Discrimination	Overall (n = 400)			Suicidal Ideation (n = 107)
	Frequency (%)	RDS-adjusted % (95% CI)	Homophily	Frequency (%^a^)^b^
**Overall**				
Never	247 (61.8)	77.6 (68.5–84.6)	1.25	33 (13.4)
In ≥ one situation	153 (38.3)	22.4 (15.4–31.5)		74 (48.4)
**At school**				
No	327 (81.8)	90.5 (84.0–94.6)	1.10	67 (20.5)
Yes	73 (18.3)	9.5 (5.4–16.0)		40 (54.8)
**Getting hired**				
No	354 (88.5)	95.2 (91.1–97.4)	1.23	75 (21.2)
Yes	46 (11.5)	4.8 (2.6–8.9)		32 (69.6)
**At work**				
No	322 (80.5)	92.2 (87.7–95.1)	1.22	56 (17.4)
Yes	78 (19.5)	7.8 (4.9–12.3)		51 (65.4)
**Getting housing**				
No	341 (85.3)	95.0 (92.3–96.8)	1.38	68 (19.9)
Yes	59 (14.8)	5.0 (3.2–7.7)		39 (66.1)
**Getting medical care**				
No	362 (90.5)	97.7 (96.0–98.6)	1.24	81 (22.4)
Yes	38 (9.5)	2.3 (1.4–4.0)		26 (68.4)
**Getting service**				
No	333 (83.3)	92.5 (88.0–95.4)	1.26	66 (19.8)
Yes	67 (16.8)	7.5 (4.6–12.0)		41 (61.2)
**In a public setting**				
No	282 (70.5)	84.0 (75.6–89.8)	1.28	45 (16.0)
Yes	118 (29.5)	16.0 (10.2–24.4)		62 (52.5)
**From police or other security personnel**				
No	285 (71.3)	85.9 (79.3–90.7)	1.44	47 (16.5)
Yes	115 (28.8)	14.1 (9.3–20.7)		60 (52.2)

*Note*. RDS = respondent-driven sampling; CI = confidence interval

^a^ Percentages are based on row frequencies.

^b^ Differences between the categories of all listed variables are significant (all p-values < .001)

### Suicidal ideation and perceived discrimination

**Fig 1:**
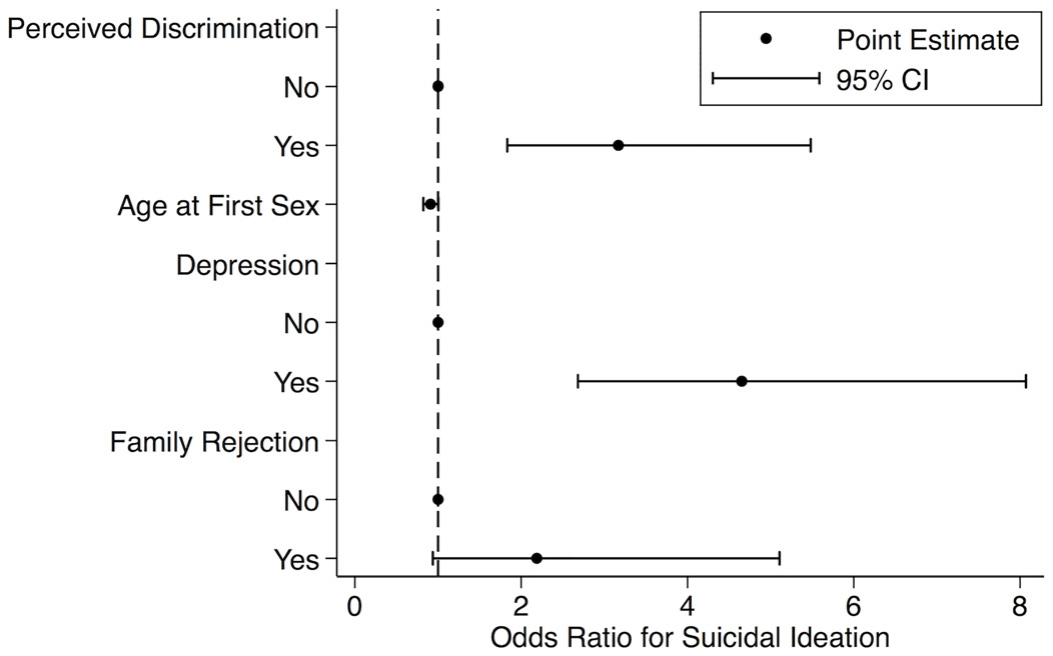
Odds ratios for suicidal ideation among MSM and TG in Nepal in 2012. Error bars indicate 95% confidence intervals.

Bivariate analysis showed that participants who had perceived discrimination on the basis of sexual orientation were more likely to have experienced suicidal ideation than participants who had never perceived discrimination on the basis of sexual orientation (48.4% vs. 13.4%, p < .001). Results of the multiple logistic regression assessing the association between suicidal ideation, perceived discrimination, and potential confounders and covariates are displayed in Table 3. In multivariable analysis, the association between perceived discrimination and suicidal ideation remained significant. Participants who had perceived discrimination on the basis of sexual orientation were around three times more likely to have experienced suicidal ideation than participants who had never perceived discrimination on the basis of sexual orientation (AOR: 3.17; 95% CI: 1.83–5.48, p < .001). The final model (Fig 1) further revealed a considerable protective effect against suicidal ideation for higher age at first sex (AOR: 0.91, 95% CI: 0.82–1.00, p = .061), a significant association between suicidal ideation and depression (AOR: 4.65, 95% CI: 2.68–8.07, p < .001), and a considerable association between suicidal ideation and family rejection (AOR: 2.18, 95% CI: 0.94–5.11, p = .070).

**Table 3:** Multiple logistic regression assessing the association between suicidal ideation, perceived discrimination, potential confounders and covariates.

Characteristic	Suicidal Ideation (n = 107)		
	n (%^a^)	OR (95% CI)	AOR^b^ (95% CI)
**Perceived discrimination**			
Never	33 (13.4)	1	1
In ≥ one situation	74 (48.4)	6.07 (3.74–9.86)***	3.17 (1.83–5.48)***
**Self-reported gender identity**			
Male	60 (21.3)	1	-
Third gender or female	47 (39.8)	2.45 (1.54–3.90)***	-
**Marital status**			
Unmarried	81 (28.0)	1	-
Married to male	7 (46.7)	2.25 (0.79–6.40)	-
Married to female	19 (20.7)	0.67 (0.38–1.18)	-
**Living situation**			
Not living with a partner	73 (24.2)	1	-
Living with male partner	24 (45.3)	2.60 (1.42–4.74)**	-
Living with female partner	10 (22.2)	0.90 (0.42–1.90)	-
**Education**			
No formal education	11 (44.0)	1	-
Primary school (1–5 years)	22 (27.5)	0.48 (0.19–1.22)	-
Secondary school and above (6+ years)	74 (25.1)	0.43 (0.19–0.98)*	-
**Occupation**			
Working	62 (25.4)	1	-
Student	14 (20.0)	0.73 (0.38–1.41)	-
Unemployed	5 (22.7)	0.86 (0.31–2.44)	-
**Age at first sex**			
Mean (SD)	15.1 (3.0)	0.86 (0.79–0.93)***	0.91 (0.82–1.00)
**Consensual first sex**			
Yes	94 (24.9)	1	-
No	13 (56.5)	3.91 (1.66–9.22)**	-
**First sexual partner**			
Male	91 (31.1)	1	-
Female	16 (15.0)	0.39 (0.22–0.70)**	-
**Transactional sex**			
No	42 (19.7)	1	-
Yes	65 (34.8)	2.17 (1.38–3.41)**	-
**HIV**			
Negative	97 (25.9)	1	-
Positive	10 (40.0)	1.91 (0.83–4.39)	-
**Depression**			
Euthymic (CES-D < 16)	24 (10.9)	1	1
Depressed (CES-D ≥ 16)	83 (46.1)	6.99 (4.17–11.70)***	4.65 (2.68–8.07)***
**SSQS**^c^			
SSQS = 6	49 (23.7)	1	-
SSQS < 6	58 (30.1)	1.39 (0.89–2.16)	-
**Family openness**			
Yes	39 (35.5)	1	-
No	67 (23.2)	0.55 (0.34–0.89)*	-
**Forced marriage**			
No	63 (22.7)	1	-
Yes	44 (35.8)	1.89 (1.19–3.01)**	-
**Family rejection**			
No	85 (23.2)	1	1
Yes	22 (64.7)	6.06 (2.88–12.75)***	2.18 (0.94–5.11)

*Note*. OR = odds ratio; CI = confidence interval; AOR = adjusted odds ratio; CES-D = Center for Epidemiological Studies Depression scale; SSQS = Social Support Questionnaire Satisfaction score.

^a^ Percentages are based on row frequencies.

^b^ The final model included perceived discrimination, depression, age at first sex and family rejection.

^c^ Cut-off point based on median-split (Skewness = -4.96, Kurtosis = 33.15).

* p ≤ .05;

** p < .01;

*** p < .001

### Suicidal ideation and the extent of perceived discrimination

**Fig 2:**
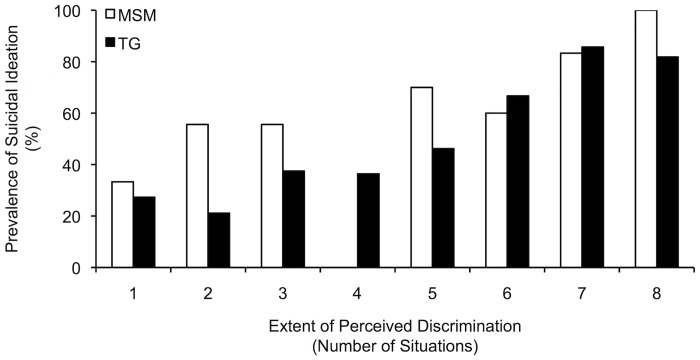
Prevalence of suicidal ideation, stratified by the number of situations in which MSM and TG had perceived discrimination on the basis of sexual orientation. MSM = men who have sex with men; TG = male to female transgender people.

Among the 153 participants who had perceived discrimination on the basis of sexual orientation, the average extent of perceived discrimination was four situations (M = 3.9, SD = 2.3). Bivariate analysis showed that participants who had experienced suicidal ideation reported more situations in which they had perceived discrimination on the basis of sexual orientation (M = 4.7, SD = 2.4), compared with participants who had never experienced suicidal ideation (M = 3.1, SD = 1.9, p < .001). The prevalence of suicidal ideation as a function of the extent of perceived discrimination is displayed in Fig 2. In multivariable analysis, the association between the extent of perceived discrimination and suicidal ideation remained significant. With each increase of one situation in the extent of perceived discrimination, participants were 35% more likely to have experienced suicidal ideation (AOR: 1.35; 95% CI: 1.15–1.60, p < .001). The final model further contained depression (AOR: 3.63, 95% CI: 1.64–8.02, p = .001).

### Attempted suicide and perceived discrimination

Bivariate analysis showed that participants who had perceived discrimination on the basis of sexual orientation were more likely to have attempted suicide than participants who had never perceived discrimination on the basis of sexual orientation (15.7% vs. 4.9%, p < .001). In multivariable analysis, the association between perceived discrimination and attempted suicide did not remain significant (AOR: 1.40; 95% CI: 0.62–3.15, p = .423). Instead, the final model revealed a protective effect for higher age at first sex (AOR: 0.77, 95% CI: 0.65–0.91, p = .003) and a significant association between attempted suicide and depression (AOR: 4.80, 95% CI: 1.94–11.87, p = .001). Furthermore, a protective effect was found for not having an immediate family member to talk to openly about the own sexual behavior (AOR: 0.36, 95% CI: 0.17–0.76, p = .007).

### Separated analysis for MSM and TG

#### Suicidal ideation and perceived discrimination

Among the 282 MSM, suicidal ideation was associated with perceived discrimination (AOR: 3.58; 95% CI: 1.76–7.30, p < .001). The final model further revealed a considerable association with age at first sex (AOR: 0.90, 95% CI: 0.80–1.01, p = .096) and a significant association with depression (AOR: 4.91, 95% CI: 2.35–10.23, p < .001). Among the 118 TG, the association between perceived discrimination and suicidal ideation did not remain significant (AOR: 1.56; 95% CI: 0.50–4.82, p = .442). Instead, the final model revealed a protective effect against suicidal ideation for being employed compared with being a student (AOR: 4.32; 95% CI: 0.93–20.18, p = .062) or being engaged in NGO work (AOR: 4.47; 95% CI: 1.37–14.57, p = .013), and a significant association with depression (AOR: 4.57, 95% CI: 1.85–11.31, p = .001) and with being forced to live outside the family’s home (AOR: 3.44, 95% CI: 1.01–11.69, p = .048).

#### Suicidal ideation and the extent of perceived discrimination

Among the 68 MSM who had perceived discrimination on the basis of their sexual orientation, the extent of perceived discrimination was the only variable associated with suicidal ideation in the final model. With each increase of one situation in the extent of perceived discrimination, MSM were 34% more likely to have experienced suicidal ideation (OR: 1.34; 95% CI: 1.05–1.72, p = .019). Among the 89 TG who had perceived discrimination on the basis of their sexual orientation, the likelihood of having experienced suicidal ideation increased by 42% with each increase of one situation in the extent of perceived discrimination (AOR: 1.42; 95% CI: 1.11–1.81, p = .005). The final model further revealed a protective effect against suicidal ideation for being employed compared with being engaged in NGO work (AOR: 4.26; 95% CI: 1.20–15.16, p = .025), and a significant association with depression (AOR: 3.51, 95% CI: 1.21–10.19, p = .021).

#### Attempted suicide and perceived discrimination

When controlling for potential confounders and covariates, no significant association was found between attempted suicide and perceived discrimination, neither among MSM (AOR: 1.78; 95% CI: 0.59–5.39, p = .305), nor among TG (AOR: 0.67; 95% CI: 0.17–2.56, p = .556). Among MSM, attempted suicide was associated with age at first sex (AOR: 0.77; 95% CI: 0.62–0.96, p = .022) and depression (AOR: 6.12; 95% CI: 1.54–24.37, p = .010). Among TG, considerable associations with attempted suicide were found for age at first sex (AOR: 0.80; 95% CI: 0.62–1.02, p = .075), and depression (AOR: 3.35; 95% CI: 0.98–11.38, p = .053). Furthermore, the final model revealed a protective effect for not having an immediate family member to talk to openly about the own sexual behavior (AOR: 0.25, 95% CI: 0.07–0.87, p = .029).

## Discussion

The results indicate a high burden of suicidality among the MSM and TG populations in Nepal. For the general population in low- and middle-income countries, prevalence estimates for suicidal ideation range between 3.1% and 12.4% for suicidal ideation [23]. In comparison, an estimated prevalence of 9.8% to 20.1% for suicidal ideation among the MSM and TG populations in Nepal seems high. Still, the rate of transition from suicidal ideation to attempted suicide is consistent with cross-national findings for the general population [23]. It further should be noted that all prevalence measures for suicidality were higher among the surveyed TG than the surveyed MSM. In addition, perceived discrimination based on sexual orientation was found to be common among the MSM and TG populations in Nepal. Discrimination from the police or other security personnel and discrimination in public settings were the most frequent forms. Again, the prevalence of perceived discrimination was higher among the surveyed TG than the surveyed MSM. An explanation for the elevated prevalence of perceived discrimination among TG might be the fact that TG are an easier target for discrimination in general. Due to their feminine gender presentation, TG are visible in society and easier to identify than MSM [16].

Previous studies have documented an association between perceived discrimination and suicidal ideation among sexual and gender minorities in high-income countries [11,12,13]. In a population-based survey from the Netherlands, gay and bisexual men who had perceived discrimination because of their sexual orientation were almost five times more likely to have experienced suicidal ideation, compared with gay and bisexual men who had never perceived discrimination, even when controlling for mental disorders [12]. The present findings further highlight perceived discrimination as an independent risk factor for suicidal ideation among sexual and gender minorities in Nepal, a low-income country. Even when controlling for potential confounders and covariates, the prevalence of suicidal ideation among the surveyed MSM was associated with perceived discrimination and increased in parallel with the extent of perceived discrimination. Among the surveyed TG, the association between the prevalence of suicidal ideation and perceived discrimination did not remain significant when controlling for potential confounders and covariates, such as depression. Nevertheless and similar to the findings among MSM, the prevalence of suicidal ideation increased in parallel with the extent of perceived discrimination. In general, these findings support the theory of the so-called building block effect, stating that exposure to stressful events is cumulative and contributes to the development of mental health problems [32].

Even though perceived discrimination and its extent was shown to be associated with suicidal ideation, no such evidence was found for an association between perceived discrimination and attempted suicide among the surveyed MSM and TG. Still, this does not necessarily mean that perceived discrimination is not related to suicide. One major limitation of using attempted suicide as a measure, for instance, is the fact that it does not include attempted suicides that were successful. However, the facts that attempted suicide remains the single most important risk factor for committed suicide [15] and that the results highlight perceived discrimination as an independent risk factor for suicidal ideation but not for attempted suicide, demand for further exploration of the role of perceived discrimination in the context of the other factors that were found to be associated with attempted suicide. The present study might be a good basis to formulate a structural equation model estimating the factors’ relative contribution to the risk of attempted suicide, for instance.

Putting the results into a broader context, it is important to note that the legislative and social environment for sexual and gender minorities is rather protective in high-income countries [12]. This might not be the case in low- and middle-income countries. In fact, more than half of all low- and middle-income countries worldwide retain repressive laws against sexual and gender minorities and prohibit sexual orientations other than heterosexual [33]. The prevalence of attempted suicide was found to be 20% higher in unsupportive environments than in supportive ones in a population-based survey from the United States [34]. Due to the fact that low- and middle-income countries are more likely to have unsupportive environments for sexual and gender minorities, one can speculate that the prevalence of correspondent discrimination and its impact on suicidality might be even higher in low- and middle-income countries than in high-income countries.

### Implications and future research

Future suicide prevention programs should be tailored to the specific needs of sexual and gender minorities and should include elements focusing on discrimination, such as information on coping strategies. In terms of political action, ongoing commitment and sustainable support regarding respect, protection and fulfillment of human rights and recognition of human rights violations is required. Furthermore, societal respect and acceptance towards sexual and gender minorities need to be established.

For enabling a successful implementation of these implications, further investigation and close collaboration with sexual and gender minorities is required. Underlying mechanisms accounting for the association between perceived discrimination and suicidal ideation need to be assessed, and moderating and mediating factors need to be identified. The association might vary according to personal coping strategies, social support, group identification, and personal characteristics [35]. Furthermore, it has to be examined whether the association is unique to perceived discrimination based on sexual orientation, or whether similar associations and interactions occur regarding perceived discrimination based on different aspects, such as race, ethnicity or immigration status [2]. Over and above, research among sexual and gender minorities in low- and middle-income countries must be scaled up. It remains unknown whether the present study’s findings can be replicated for sexual and gender minorities other than MSM and TG, and in low- and middle-income countries other than Nepal.

### Strengths and limitations

The present study is among the first to explore the role of perceived discrimination for suicidal ideation among sexual and gender minorities in a low-income setting. Further, it shows methodological strengths. First, a large sample of MSM and TG was located through RDS, a sampling method that has been shown to provide unbiased population estimates when the criteria are fulfilled [36]. In contrast, representative population-based samples are often criticized for the small proportion of sexual and gender minorities included and for combining data from different minorities to increase power [37], while snowball sampling is often criticized for producing biased samples through tending to oversample participants with a larger social network [38]. Second, using data from a large-scale surveillance survey allowed accounting and adjusting for various potential confounders and covariates.

Despite these strengths, certain limitations need to be considered. First, the cross-sectional nature of the data does not allow causal inferences to be drawn. According to established theories, suicidal ideation is a consequence of perceived discrimination [39,40]. On the other hand, suicidal ideation could reduce social functioning and lead to discrimination in the first place, or pre-existing suicidal ideation might simply lead to an increased likelihood to interpret negative events as discrimination [11]. Second, the findings are based on self-reported measures, which may lead to the emergence of different biases, such as recall biases or response biases related to social desirability [41]. Self-reported discrimination, in particular, might be biased for several reasons: due to forms of internalized oppression, such as internalized heterosexism wherein homophobic attitudes and assumptions present in society are internalized and related discrimination is accepted and thus not reported [42]; due to attributing discrimination to sexual orientation even though it might be based on different aspects, such as race or ethnicity; or due to interpreting negative events as discrimination in the first place. Although these biases can lead to over- or under-reporting of objective discrimination, it is important to keep in mind that the mere perception of discrimination is a form of stress with a potential influence on health, regardless of its objective presence [35]. Third, using data from a large-scale surveillance survey comes with certain limitations: different time frames were used for different measures and most measures were based on a limited number of items. Suicidality, for instance, was only based on three items and no data were obtained on reliability or validity. Furthermore, the measures did not cover other indicators for suicidality, such as hopelessness and burdensomeness [43], or psychache [44], and did not differentiate between participants who had never experienced discrimination and participants who had experienced discrimination but based on aspects other than sexual orientation.

### Conclusion

A high burden of suicidality is prevalent among sexual and gender minorities in Nepal and positively associated with perceived discrimination on the basis of sexual orientation and its extent. Future suicide prevention programs should target sexual and gender minorities and should include elements focusing on discrimination. Furthermore, the findings demand that political action be taken against discrimination on the basis of sexual orientation.
